# Isolation of *Clostridium perfringens* Type B in an Individual at First Clinical Presentation of Multiple Sclerosis Provides Clues for Environmental Triggers of the Disease

**DOI:** 10.1371/journal.pone.0076359

**Published:** 2013-10-16

**Authors:** Kareem Rashid Rumah, Jennifer Linden, Vincent A. Fischetti, Timothy Vartanian

**Affiliations:** 1 Tri-Institutional M.D.-Ph.D. Program of Weill Cornell Medical College, Rockefeller University and Memorial Sloan-Kettering Hospital, New York, New York, United States of America; 2 The Brain and Mind Research Institute and the Department of Neurology, Weill Cornell Medical College, New York, New York, United States of America; 3 The Laboratory of Bacterial Pathogenesis and Immunology, Rockefeller University, New York, New York, United States of America; University of Jaén, Spain

## Abstract

We have isolated *Clostridium perfringens* type B, an epsilon toxin-secreting bacillus, from a young woman at clinical presentation of Multiple Sclerosis (MS) with actively enhancing lesions on brain MRI. This finding represents the first time that *C. perfringens* type B has been detected in a human. Epsilon toxin’s tropism for the blood-brain barrier (BBB) and binding to oligodendrocytes/myelin makes it a provocative candidate for nascent lesion formation in MS. We examined a well-characterized population of MS patients and healthy controls for carriage of *C. perfringens* toxinotypes in the gastrointestinal tract. The human commensal *Clostridium perfringens* type A was present in approximately 50% of healthy human controls compared to only 23% in MS patients. We examined sera and CSF obtained from two tissue banks and found that immunoreactivity to ETX is 10 times more prevalent in people with MS than in healthy controls, indicating prior exposure to ETX in the MS population. *C. perfringens* epsilon toxin fits mechanistically with nascent MS lesion formation since these lesions are characterized by BBB permeability and oligodendrocyte cell death in the absence of an adaptive immune infiltrate.

## Introduction

How MS begins remains unknown. The earliest lesions studied, fixed hours after onset of symptoms, exhibit blood-brain barrier (BBB) permeability, oligodendrocyte apoptosis, and early microglial activation [1]–[3]. In these nascent lesions, demyelination is not yet apparent, there are no lipid-laden macrophages and there is the conspicuous absence of infiltrating lymphocytes [1]–[6]. The absence of an inflammatory infiltrate in nascent lesions argues against MS beginning as an autoimmune phenomenon and instead favors a toxin or viral etiology. We reasoned that the environmental trigger for initial lesion formation in MS might be a soluble toxin based on the histopathologic features of the nascent lesion.

*C. perfringens* is a gram positive, spore forming anaerobe that is sub-categorized into five toxinotypes based on combinatorial carriage of α, β, ε and ι toxins [7], [8]. *C. perfringens* types B and D carry the ETX gene, which encodes a 33 kD protoxin [8]–[10]. With log phase growth, protoxin is secreted and cleaved by trypsin and chymotrypsin in the gastrointestinal (GI) tract or by the *C. perfringens* encoded λ-protease, yielding an active toxin which is ∼1,000X more potent than the protoxin [8], [10].

The natural hosts for *C. perfringens* toxinotypes B and D are ruminant animals in whom ETX-mediated neurologic symptoms occur when carbohydrate rich feed or over grazing favors exponential growth of the bacilli [11]–[15]. ETX is absorbed via the intestine [11], [14], [16]–[18], enters the blood stream and permeabilizes the BBB, resulting in MS like symptoms (e.g. visual dysfunction, incoordination and spastic paralysis) [8], [10]. Murrell and colleagues, because of these effects on the CNS [19], first suggested ETX as a potential MS trigger although humans are not natural hosts for *C. perfringens* types B or D [7], [8], [20]–[22].

ETX binds to an unknown receptor present both in the brain vasculature and myelinated brain regions e.g. corpus callosum [10], [23]–[26]. Once bound to its receptor, ETX integrates into the plasma membrane as a heptameric pore, leading to osmolysis [27]–[31]. When ETX is administered to rodents, BBB disruption occurs and white matter vasculature is especially vulnerable [32]–[36]. Interestingly, intraperitoneal administration of protoxin in rats results in the formation of focal ovoid lesions within the corpus callosum, in which the long axis of the ovoid is oriented perpendicular to the surface of the lateral ventricle [36]. Dawson first described this specific lesion morphology and the radiographic equivalent is all but pathognomonic for clinically definite relapsing remitting multiple sclerosis [37]. We postulate that *Clostridium perfringens* epsilon toxin may be a candidate causative toxin for nascent lesion formation in MS worthy of further investigation.

## Methods

### Ethics Statement

Research protocol #1003010940 for the collection of samples from individuals with MS and healthy controls was reviewed and approved by the WCMC institutional review board. All participants in the study gave written informed consent.

### Fluorescent Labeling of ETX and Immunofluorescence

His tagged protoxin was procured from BEI Resources and 1 mg was fluorescently labeled using Alexa Fluor 594 Protein labeling Kit (Invitrogen) as per manufacturer’s instructions.

#### Retina

Fresh frozen tissue sections were incubated with BSLI (Vector Labs) 1∶200, and Alexa 594 labeled His-tagged protoxin (50 nM) for 1 hr at RT. After three 5 minute washes in PBS, stained sections were post fixed in 4% PFA for 10 mins at RT. The stained tissue was washed 3X in PBS, mounted and imaged.

#### Brain

Fixed frozen coronal brain sections were permeabilized in a 1% sodium cholate, 1% BSA, 10% donkey serum, PBST solution overnight at 4 degrees C. Sections were then incubated with rabbit anti-PLP (ThermoScientific) at 1∶1000 overnight at 4 degrees C. Following three washes with PBS, sections were then incubated with Donkey anti-rabbit Alexa 488 (Jackson ImmunoResearch) at 1∶1000, and Alexa 594 labeled His-tagged protoxin (50 nM) for 2 hrs at RT. The stained tissue was washed 3X in PBS and prepared for microscopy at the Rockefeller Bio-Imaging facility.

### Sample Collection/Fecal Culture/PCR Analysis

Stool specimens were self-collected by patients and healthy controls in a clean single use vessel and stored at −20 degrees C until returned to the MS Center. Approximately one gram of stool was collected and stored in a fecal collection tube (Sarstedt) containing 9 ml of buffered glycerin-salt solution (10% glycerin, 71.2 mM K_2_HPO_4_, 29.4 mM KH_2_PO_4_, 71.9 mM NaCl made in distilled water, adjusted to pH 7.2 and autoclaved) under IRB protocol no. 1003010940. Upon receipt, samples were resuspended in 40 ml of modified rapid perfringens media (RPM) [38]; D-cycloserine (400 mg/L) was substituted for neomycin/polymyxin B and litmus milk was omitted to improve DNA extraction. The resuspended samples were cultured in 50 ml falcon tubes with tightly closed caps at 47 degrees C ON.

DNA was extracted was from 1 ml of culture supernatant using a Qiagen blood and tissue kit. Isolated DNA was used as template for the following PCR reactions; the following primers were used:

1. 16S rRNA (positive control) fwd primer: AGAGTTTGATCCTGGCTCA, reverse primer: GGTTACCTTGTTACGACTT
2. Alpha toxin (pan *C. perfringens* marker) fwd primer: GCTAATGTTACTGCCGTTGA [39], reverse primer: CCTCTGATACATCGTGTAAG [39]
3. Beta toxin fwd primer: GCGAATATGCTGAATCATCTA [39], reverse primer: GCAGGAACATTAGTATATCTTC [39]
4. Epsilon toxin fwd primer: GCGGTGATATCCATCTATTC [39], reverse primer: CCACTTACTTGTCCTACTAAC [39]
5. B1RB**B5** phage gene fwd primer: AAATGGACAAGAGGGATAAGGAT, reverse primer: TTTTCATCACAAATACCAGCCTC
6. B1RA**A6** phage gene fwd primer: TTACAATAAAACCACATGAGCTT, reverse primer: TTTTATTTAACATACTCCGTTTT
7. Q8SB**N7** phage gene fwd primer: GGGTGTCAAAGAAGATTTTAAAG, reverse primer: TTCTATCTTGCAACATTATATTT.

### Isolation and Characterization of *C. Perfringens* from Patient Fecal Sample

Approximately 0.5 g of the patient’s frozen fecal sample was resuspended in 10 mls of PBS. To select for vegetative cells, 5 mls of the resuspended fecal sample were inoculated into 20 mls of RPM. To select for *C. perfringens* spores, the remaining 5 mls of resuspended fecal sample were heat shocked at 85 C for 15 minutes before inoculation into RPM. Fecal RPM cultures were incubated overnight at 37 degrees C. The following morning, 100 ul of the overnight fecal RPM cultures were sandwiched between two layers of Perfringens Agar Base (Oxoid) supplemented with 400 mg/L D-cycloserine (Sigma) to make egg yolk free Tryptose Sulfite Cycloserine (TSC) agar. 10 mls of molten TSC were used to coat the bottom of a petri dish and allowed to solidify. 100 ul of the fecal RPM culture were spread evenly over the hardened 10 mls of TSC and allowed to adhere for 10 minutes at room temperature. 25 mls of cooled but still molten TSC agar was then layered on top, allowed to solidify, and TSC sandwich plates were incubated at 37 C. Black colonies characteristic of *C. perfringens* were plucked from the TSC agar and inoculated into RPM and incubated at 37 degrees C overnight. To isolate pure colonies, RPM subcultures were streaked onto Schaedler Agar with Vitamin K1 and 5% Sheep Blood (BD) and anaerobically incubated (BD GasPak EZ system) at 37 degrees C overnight. Colonies exhibiting *C. perfringens* like morphology and beta hemolysis were isolated by repeat subculture into RPM and streaking onto Schaedler blood agar plates until a pure colony was achieved. Genotype was confirmed by PCR as previously described [39]. Growth curves were generated by measuring optical densities of cells at 600 nm (OD 600) using a Biophotometer plus (eppendorf). Overnight cultures were diluted to an OD600 of 0.1 in RPM, incubated at 47 degrees C, and OD readings measured at indicated time points. ATCC 3626 strain was used as the laboratory type B strain. All strains were maintained on TSC agar or Shaedler blood agar.

### Serum and CSF Collection

CSF and sera from people with MS and healthy controls were obtained from banked samples at the Weill Cornell MS Center. Additional MS and Stroke CSF and sera were obtained from the Brain Research Institute, UCLA. SLE sera were purchased from Vital Products Inc. Disease ascertainment for the Cornell samples was made by an MS specialist and was based on Poser or McDonald criteria. Information on disease activity, disease duration, MRI activity, genetics, medications and other relevant medical history or lifestyle questions was unavailable for the serum and CSF samples.

### Statistical Analysis

A two-tailed Chi squared test was performed to compare the prevalence of commensal *Clostridium perfringens* type A and anti-ETX immunoreactivity in MS patients vs. healthy controls.

### ETX Immunoreactivity

Western blots were performed using human sera or CSF as primary antibody. SDS page electrophoresis was run and each well was loaded with a mixture of 100 ng of His tagged proETX (BEI Resources) and molar equivalents of PA63 (EMD Millipore) 190 ng, Cholera toxin beta FITC (Sigma Aldrich) 36 ng, His tagged Shiga toxin 1 beta (BEI Resources) 26 ng and His tagged Shiga toxin 2 beta (BEI Resources) 26 ng. Proteins were transferred to an Immobilon P membrane (Millipore) and probed with diluted sera/CSF. All serum and CSF samples were diluted 10,000 fold and 27 fold respectively, while SLE sera were diluted 100, 000 fold to normalize background. HRP conjugated Donkey anti-human IgG 1∶10,000 (Jackson Immunoresearch) was used to visualize human antibody binding.

## Results

### Identification of *Clostridium Perfringens* Type B in a Woman with Multiple Sclerosis

**Figure 1:**
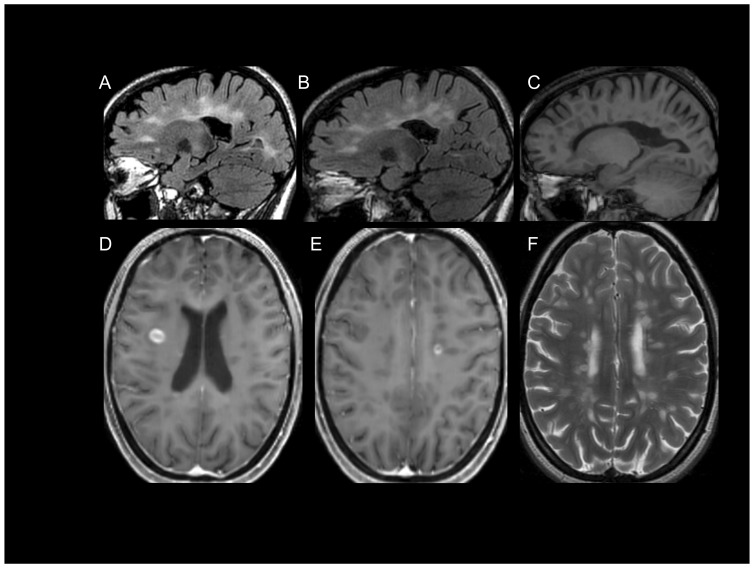
Brain MRI of patient 73F. (A) FLAIR image from September 2011 showing characteristic lesions in a parasagittal plane. (B–F) MRI from May 2012 revealing characteristic lesions on FLAIR imaging as before (B), T1 hypointensities (C), characteristic contrast enhancing lesions post IV Gadolinium (D, E), and characteristic lesions on T2 weighted axial image (F).

A 21-year old woman (patient 73F) developed left lower extremity dyscoordination, and imbalance that evolved to its maximum deficit over three days. Two weeks after onset she was referred to a neurologist due to persistent symptoms and neuroimaging of the brain revealed multiple foci of increased T2/flair signal in the deep and subcortical white matter, with several ovoid lesions within the corpus callosum characteristic of MS [40]–[42]. Following administration of IV gadolinium, several lesions enhanced. CSF analysis revealed five IgG bands on isoelectric focusing that were not present in the corresponding serum sample. She met revised criteria for clinically definite relapsing remitting MS at the earliest clinical presentation termed a clinically isolated syndrome (CIS) [43]. She received five days of IV methylprednisolone, 1 gram per day, and her symptoms resolved to normal neurological function within three weeks. She was referred to the Weill Cornell MS Center for confirmation of diagnosis and treatment planning. Repeat neuroimaging at Weill Cornell revealed lesions characteristic in morphology and location for Multiple Sclerosis (Figure 1A). Approximately three months after onset of symptoms, she was enrolled in the HITMS (**H**arboring the **I**nitial **T**rigger of **M**ultiple **S**clerosis) study, IRB protocol no. 1003010940, and a self-collected stool sample was obtained. Disease modifying treatment was initiated. Eight months after initiation of treatment she remained asymptomatic and her first treatment assessment MRI was performed which revealed several new contrast enhancing lesions (Figure 1B–F).

**Figure 2:**
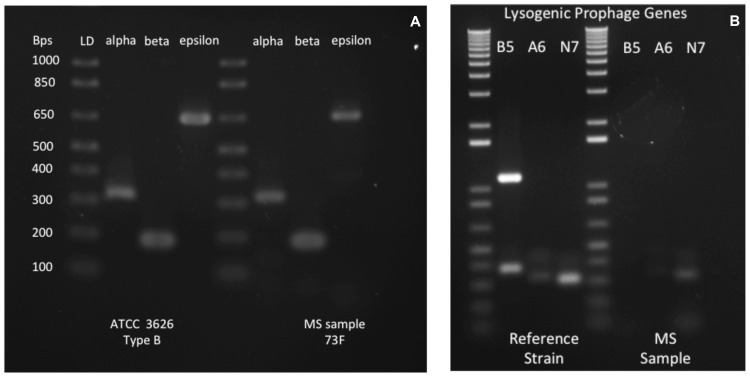
*C. perfringens* type B in a woman with RRMS, and the prevalence of *C. perfringens* type A in MS and healthy controls. (A) Left panel shows PCR based genotyping of ATCC 3626 type B strain and from patient 73F. PCR products for α, β, and ε toxin are identified in both. (B) To exclude the possibility that the type B strain identified in the stool of patient 73F was a contaminant, the profile of lysogenic prophage genes was determined in the laboratory strain and in the patient isolate (right panel). ATCC 3626 reference strain possesses all three prophage insertions, whereas the patient’s strain possesses only the A6 (weakly) and N7 prophage insertions. Phage genes and PCR product size: B1RBB5, 1000 bps; B1RAA6, 300 bps; Q8SBN7, 300 bps.

Three months after onset of her first symptoms, patient 73F was found to harbor *C. perfringens* type B in her GI tract. PCR analysis revealed carriage of genes encoding α, β, and ε toxins (Figure 2A). This represents the first human known to carry type B and the first MS patient found to carry an ETX producing *C. perfringens.* To exclude a possible laboratory-derived contaminant, we performed a lysogenic bacteriophage footprint analysis of the laboratory (ATCC 3626) and patient-derived *C. perfringens* strains. Three lysogenic bacteriophage insertions were identified in the laboratory strain, which matched the known whole genome sequence (Figure 2B). The patient’s strain contained just two lysogenic bacteriophage insertions, thus confirming that the patient-derived ETX amplicon was not a laboratory contaminant (Figure 2B). Since a combination of toxinotypes C and D would also result in identification of α, β, and ε toxin genes, we sequenced the patient-derived ETX gene confirming that it was derived from a type B ETX plasmid [44].

**Figure 3:**
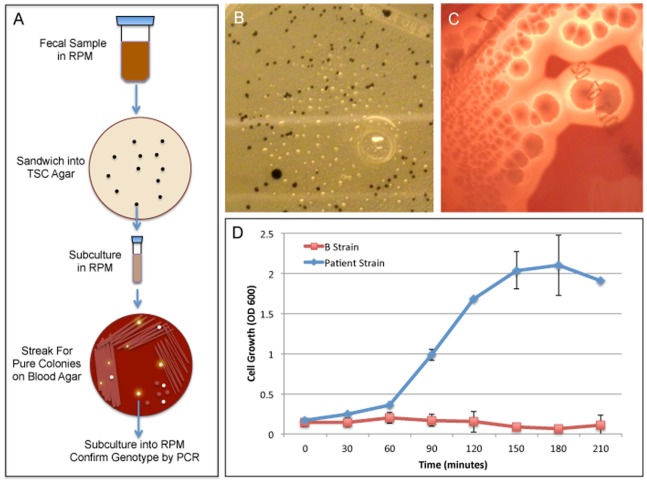
Isolation and Characterization of a patient-derived *C. perfringens* type B strain. (A) Schematic of the isolation procedure described in methods section. Briefly, the patient’s fecal sample was resuspended in RPM, incubated overnight at 37C, and inoculated into TSC sandwich plates. Colonies with characteristic *C. perfringens* morphology (black colonies) were subcultured into RPM and then streaked onto Schaedler blood agar plates until pure colonies with characteristic *C. perfringens* morphology that exhibited beta hemolysis were isolated. Strain genotype was confirmed by PCR analysis for toxin genes. (B) Typical black *C. perfringens* like morphology in TSC agar from patient 73F diluted fecal RPM sample. (C) Typical *C. perfringens* like morphology on Schaedler blood agar plates exhibiting beta hemolysis from a subcultured TSC colony from patient 73F. (D) Cell growth of laboratory B strain (Lab B Strain) compared to the patient-derived B strain (Patient B Strain) at 47 C measured by optical density at 600 nm (OD 600) at the given time points.

To further prove that the detection of a *C. perfringens* type B from patient 73F was indeed a unique strain, we generated pure colonies for analysis. Liquid RPM cultures of patient 73F’s fecal samples were sandwiched into TSC agar. TSC agar contains sodium metabisulphite and ferric ammonium citrate, which act as indicators of sulphite reduction by turning black. Because *C. perfringens* are sulfite-reducing bacteria, black colonies from the sandwich TSC plates were plucked (Figure 3B), subcultured in RPM and streaked onto Schaedler blood agar plates. Colonies that exhibited characteristic *C. perfringens* morphology and beta hemolysis (Figure 3C) were picked and subcultured until pure colonies were obtained. Strain genotype was confirmed by PCR. Both the laboratory (ATCC 3626) *C. perfringens* type B strain showed robust growth at 37 C (data not shown). Importantly, the laboratory strain failed to grow at 47 C (Figure 3D) whereas the patient-derived type B strain showed robust growth at 47 C. In conjunction with the lysogenic bacteriophage footprint, this data confirms that our patient-derived stain is unique and not a laboratory contaminant.

### Reduced Prevalence of *C. Perfringens* Type A in MS Compared to Healthy Controls

We assessed the prevalence of *C. perfringens* type A, a human commensal, in MS and healthy controls. Stool samples were prospectively collected for analysis; 31 healthy controls and 30 MS subjects were studied. The mean age of healthy controls was 46.7 years (range: 22–64 years) and the mean age of MS subjects was 42.0 years (range: 21–58 years). Of the healthy controls, 14 of 31 were female, and of the MS subjects 22 of 30 were female. Disease classification for the 30 MS subjects: 26 relapsing remitting; and 4 secondary progressive. The mean Expanded Disability Status Scale (EDSS) score for the MS subjects at time of enrollment was 1.91 with a range of 0–5.0.

**Figure 4:**
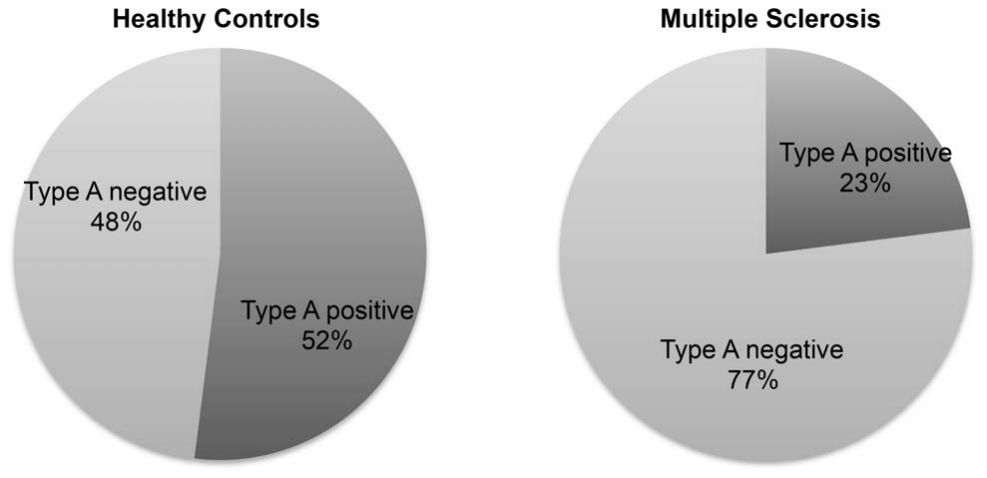
The prevalence of *C. perfringens* type A, a human commensal, was determined in MS patients and healthy controls. Culture of stool in *C. perfringens* compatible growth medium revealed that 52% of health controls harbor *C. perfringens* type A in the gastrointestinal tract, whereas only 23% of people with MS harbor type A.

Bacteria were lysed, DNA isolated and toxinotypes determined by PCR analysis [39]. Prior published studies have demonstrated that type A is present in approximately 50% of healthy humans [45]. Consistent with this, we found that 52% of the healthy controls (n = 31) carried detectable type A (Figure 4). However, we found only 23% *C. perfringens* type A carriage in individuals with MS (n = 30), χ^2^p = 0.0227 (Figure 4).

### Frequency of Immunoreactivity to Epsilon Toxin in MS and Healthy Controls

**Figure 5:**
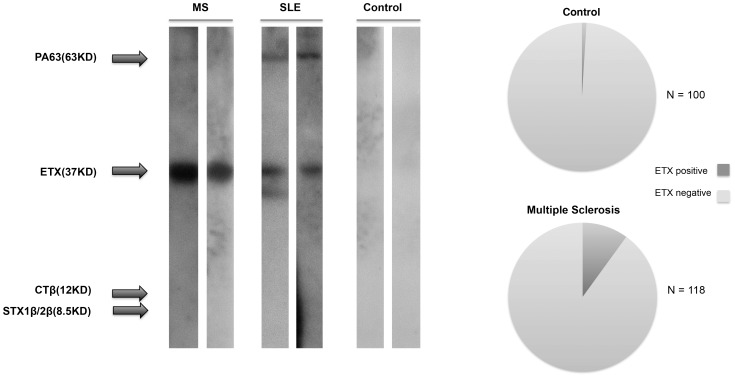
Immunoreactivity to ETX in people with MS, SLE and healthy controls. Left panel shows Western blots. The two MS blots shown are characteristic for true positives: immunoreactivity to the *C. perfringens* proETX protein at 37 kD but not to the other toxins present on the blot including PA63 at 63 kD. The two blots probed with SLE sera are characteristic of false positives in that immunoreactivity is also present for PA63. Controls shown are true negatives with no immunoreactivity to any of proteins present on the blot. Note that the proETX gene encodes a protein with a predicted MW of 33 kD, which runs on SDS-PAGE with an apparent MW of 37 kD. The right panel shows prevalence of immunoreactivity to ETX in serum and/or CSF of people with MS and healthy controls.

We screened sera and CSF from MS patients, healthy controls and other diseases for immunoreactivity to ETX by Western blot using the proETX protein. Sera and CSF were obtained from three sources of banked samples as described in the materials and methods. We developed a Western blot assay that rigorously excluded the likelihood of false positives. A sample was scored positive if there was clear immunoreactivity for ETX in conjunction with no immunoreactivity to four control toxins. Three of the controls were chosen to represent known gut-derived toxins: Cholera toxin beta, Shiga toxin 1 beta and Shiga toxin 2 beta; no cross-reactions ever occurred with these control toxins. A fourth control, protective antigen 63 (PA63) from *Bacillus anthracis* was chosen because like ETX, PA63 is a pore-forming toxin with a hydrophobicity map similar to epsilon toxin [46]–[49]. PA63 was also chosen because most humans should be seronegative. Seroreactivity to PA63 would occur only in instances of vaccination or exposure to Anthrax. Most humans are not vaccinated against Anthrax and in our study; none of the patients or controls was vaccinated. Seroreactivity to PA63 could also be observed in people who have been infected with *Bacillus anthracis* and survived. Since pulmonary and gastrointestinal Anthrax is usually fatal or debilitating, and since cutaneous Anthrax results in a characteristic black eschar, it is unlikely that prior Anthrax would be missed on a directed health questionnaire [50]–[52]. Thus, positive immune reactivity to PA63 would strongly suggest non-specific interaction of host antibodies with PA63 or prior exposure to an antigen with a shared epitope. We thus excluded samples that showed immunoreactivity against ETX and PA63 since these indicated equivocal results. In SLE, where there is heightened humoral immunity, cross-reactions were common (Figure 5A). Since hydrophobic proteins are more likely to show non-specific interactions with antibodies, we favor the idea that immunoreactivity to PA63 is nonspecific in nature.

We found that 10% of MS patients and 1% of controls, χ^2^p = 0.0044, in a cross-sectional analysis, possessed ETX specific antibodies (Figure 5). Based on the known low rates of seropositivity following immunization, and the common seroreversion rates [53], these rates in MS and controls presumably underestimate the true value of ETX exposure.

Data shown in Figure 5 are the combined CSF and serum results. The CSF and serum samples were from different patients and thus there is no overlap in the data analysis. For CSF, 6 of 62 MS patients, and 1 of 40 controls, were positive for ETX immunoreactivity. For the analysis of sera, 6 of 56 MS patients, and 0 of 60 controls were seroreactive to ETX. While we did not have matching serum samples for the CSF samples, we did have matching plasma samples for each of the CSF samples. To determine if all the patients who had positive immunoreactivity to ETX in their CSF also harbored immunoreactivity in their blood, we examined matching CSF and plasma samples. The plasma immunoreactivity to ETX mirrored exactly that of the CSF; i.e. of the 6 patients who showed ETX immunoreactivity in their CSF, all showed immunoreactivity to ETX in their plasma. Similarly, all of the CSF ETX negative patients were also negative in their plasma.

### Specificity of *C. Perfringens* Epsilon Toxin for CNS Endothelial Cells and White Matter

**Figure 6:**
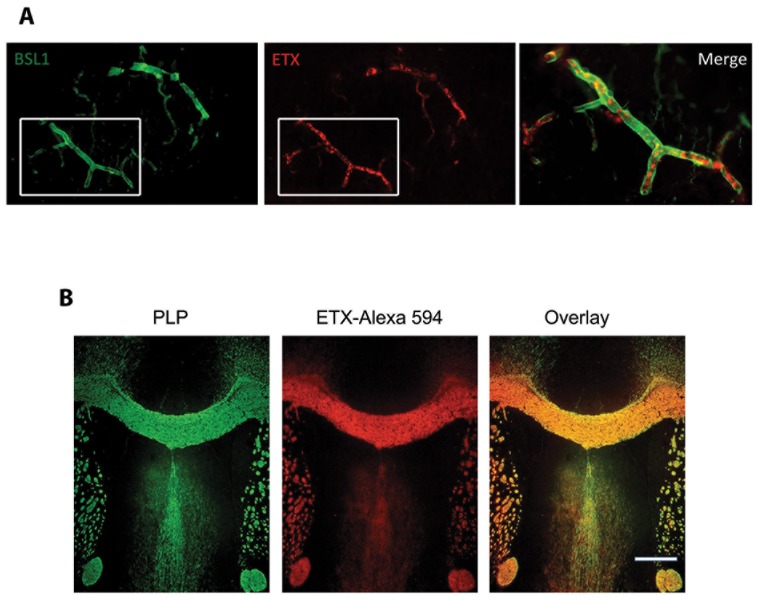
ETX binds specifically to retinal vessels and myelin. A) Frozen sections of adult mouse retina were stained with pan-vessel marker FITC labeled BSL1 (green) and Alexa 594 labeled ETX (red). A merge and enlargement of the white boxes show co-labeling of a retinal vessel. B) Fixed frozen coronal sections from adult mouse brain through the corpus callosum were stained for proteolipid protein (PLP, green), and Alexa 594-ETX (red). Intense staining with ETX is observed in all PLP-positive white matter tracts. Merged PLP and ETX images reveal essentially complete overlapping fluorescent signal. Bar = 500 µm.

ETX binding to endothelial cells within brain vasculature and to white matter has been previously reported [25], [32], [33], [54]. We verified ETX specificity for white matter and examined staining of retinal vessels by ETX. The retina is embryologically part of the CNS and retinal vessels possess a blood-retinal barrier analogous to the blood-brain barrier. Interestingly, retinal phlebitis or retinal vasculitis is a common yet unexplained observation in MS patients [55], [56]. Furthermore, recent studies using high-resolution optical coherence tomography have identified microcystsic macular edema of unknown etiology in MS, which is an indication of focal blood-retinal barrier damage [57]–[60]. We probed frozen sections of adult mouse retina with fluorescently tagged ETX and found that ETX colocalized to retinal vessels (Figure 6A). To verify that ETX binding in the CNS localizes to myelin, we fluorescently labeled ETX to stain mouse brain cryosections. Myelin was identified by immunoreactivity against the proteolipid protein (PLP). ETX binding in the mouse CNS shows essentially complete overlap with CNS myelin (Figure 6B). Taken together with prior published reports of ETX binding to brain vasculature and disruption of the BBB, binding of ETX to white matter presents a clear mechanistic link between ETX, BBB disruption and oligodendrocyte/myelin injury.

## Discussion

Kurtzke and Hyllested reported a detailed analysis of MS epidemics on the Faroe Islands [61]–[64]. Prior to 1943, there were no documented cases of MS on the Faroes, an impressive absence considering that neighboring Iceland, Sweden and Denmark each reported a high annual MS incidence [65], [66]. With the common Norse ancestry of the Faroese, Icelandic, Swedish and Dane peoples, the absence of MS in the Faroes prior to 1943 strongly suggests the existence of an environmental initiator. During World War II, coincident with the arrival of British troops, the first of four documented MS epidemics within native Faroese was reported [61]–[66]. Since haplotypes are undoubtedly stable in that short time period, this information further substantiates that MS requires an environmental trigger. Kurtzke also identified a co-incident rise in gastrointestinal infections following British military occupation, and postulated the trigger to be a pathogen spread by fecal-oral transmission [67]. T.C.G. Murrell noted that the prevalence of MS was high in regions where sheep were concentrated and raised the possibility that ETX or other sheep associated pathogens may be responsible for causing MS [19].

Sir Bradford Hill described nine criteria for determining if an environmental agent is causally associated with a disease [68]–[70]. One of these criteria is *Mechanistic Plausibility*, i.e. do the known actions of the vector/agent theoretically fit with what is known about the disease. The question then is, what do we know mechanistically about how MS lesions initially form. An optimal approach for generating mechanistic hypotheses regarding how new lesions form in MS is examination of lesions at their onset. Relatively few studies have analyzed pathologic specimens containing lesions 1–2 days old. In Prineas’ analysis of nascent lesions, he observed oligodendrocyte apoptosis with preservation of myelin, blood-brain barrier (BBB) disruption and early microglial activation [1]–[3]. These lesions may reflect the pathologic equivalent of what Lucchinetti and colleagues refer to as Type III lesions [71], [72]. With an absence or paucity of lymphocytes, nascent [1]–[3] or Type III lesions [71], [72] are postulated to arise from an environmental insult such as a toxin or virus targeting oligodendrocytes. Since *C. perfringens* epsilon toxin targets CNS microvascular endothelial cells and oligodendrocytes [25], [32], [33], [54], and since we find that there is a 10-fold increase in immunoreactivity to epsilon toxin in MS patients versus controls, we raise the hypothesis that *C. perfringens* epsilon toxin is a candidate disease and lesion initiating environmental agent in MS.

Immunoreactivity to ETX was identified in about 10% of people with MS and 1% of healthy controls. The low value of immunoreactivity to ETX may be explained by the difficulty that mammals have sustaining humoral immunity to ETX [53]. For example, when vaccinated at t = 0 and t = 6 weeks with epsilon toxoid, only 50% of goats have protective anti-toxin titers at week nine [53]. By week 30, at the time of the 3^rd^ vaccination only 2% of the goats maintain protective titers. At week 32 (2 weeks after the 3^rd^ vaccination), 100% have protective titers, but by week 56 only 11% show protective titers [53]. Thus, in mammals exposed to epsilon toxin, seronegativity and seroreversion are common even when the toxin is administered with an adjuvant. We thus postulate that the values we obtained from healthy controls and MS subjects for ETX immunoreactivity are likely underestimating the true incidence of ETX exposure.

We find that people with MS are less likely to harbor *C. perfringens* type A when compared to controls. Soil studies have identified that the presence of *C. perfringens* type A is coincident with the absence of other toxinotypes, suggesting that toxinotype A may compete with other *C. perfringens* toxinotypes for resources [73]. While the type A toxinotype may outcompete *C. perfringens* types B and D within an ecological niche, there are other factors that could contribute to, or account for the observed difference in *C. perfringens* carriage. Important considerations are host genetics, diet, use of probiotics, medications, gut microbiota, and use of antibiotics. In this study, none of the subjects received cytotoxic or immune suppressing agents. Furthermore, none of the subjects had undergone antibiotic treatment of any kind in the six months prior to sample collection, or greater than two weeks of antibiotics in the two years prior to sample collection. Host genetics and diet were not assessed in this study. The absence of *C. perfringens* type A may open a theoretical ecological niche for *C. perfringens* types B or D, but its absence is not tantamount to the presence of these toxinotypes.

We identified one case in which a newly diagnosed patient harbored *C. perfringens* type B. Eight months after initially testing positive for *C. perfringens* type B, she remained positive for toxinotype B upon repeat analysis (data not shown). However, we expect that identification of *C. perfringens* types B or D in humans will be difficult, as *C. perfringens* forms endospores that are resistant to standard DNA extraction methods. Additionally, the organism is likely to exist in low abundance in the upper GI tract, only rarely entering growth phases that render it detectable.

Although ETX binds to peripheral nervous system (PNS) myelin, as it does CNS myelin [25], autoradiograph studies show that ETX only targets the CNS and not the PNS [74]. We propose that ETX fails to bind to PNS endothelial cells that comprise the blood-nerve barrier; therefore PNS myelin is not exposed to the toxin.

Finally, binding of ETX to retinal veins that form the blood-retinal barrier (BRB), a CNS barrier analogous to the BBB, may explain the enigmatic observation of periphlebitis retinae in people with MS [55], [56]. The human retina is typically devoid of myelin, yet vascular scarring occurs [75]. Primary ETX action on the BRB may result in retinal phlebitis that is independent of oligodendrocytes or myelin. Furthermore, serum protein leakage and the accumulation of perivenular monocytes in the absence of oligodendrocyte apoptosis or demyelination are often observed in pathologic MS brain specimens [6]. These observations may similarly be due to subtle insult of the endothelium and a secondary innate immune response.
